# Rat Sarcoma (RAS)-Protein-Targeting Synthetic Cell-Penetrating Peptide as an Anticancer Biomaterial

**DOI:** 10.34133/bmr.0175

**Published:** 2025-04-15

**Authors:** Gookjin Yoon, Jinsook Suh, Beom Soo Jo, Dong Woo Lee, Deogil Kim, Moonsil Choi, Eui Kyun Jeong, Hoo Cheol Lee, Hye Min Shin, Yu-Bin Kim, Sanghui Seok, Yoon Shin Park, Chong Pyung Chung, Jue-Yeon Lee, Yoon Jeong Park

**Affiliations:** ^1^ Department of Dental Regenerative Biotechnology and Dental Research Institute, School of Dentistry, Seoul National University, Seoul 03080, Republic of Korea.; ^2^ Research Institute, Nano Intelligent Biomedical Engineering Corporation (NIBEC), Seoul 03127, Republic of Korea.; ^3^ Department of Biological Sciences and Biotechnology, School of Biological Sciences, College of Natural Sciences, Chungbuk National University, Cheongju 28644, Republic of Korea.; ^4^ School of Dentistry, Seoul National University, Seoul 03080, Republic of Korea.

## Abstract

Various bioactive materials, including peptides, have become potential candidates for slowing cancer growth and metastasis. Among bioactive peptides, a synthetic cell-penetrating peptide referred to as rat sarcoma (RAS)-binding peptide (RBP) was suggested as a potential entity that targets RAS with high affinity in MDA-MB-231 cancer cells. This RAS binding further inhibits the RAS–rapidly accelerated fibrosarcoma (RAF) protein–protein interaction. The current study revealed that RBP effectively suppresses proliferation and extracellular signal-regulated kinase 1/2 (ERK1/2) phosphorylation by disrupting the RAS–RAF interaction. This intervention not only inhibits cell migration and invasion but also has substantial potential for preventing metastasis. The RAS–RAF–ERK1/2 pathway is a key target for anticancer drug development because of frequent ERK and mitogen-activated protein kinase activation in human cancers. MDA-MB-231, a triple-negative breast cancer cell line, harbors a G13D Kirsten rat sarcoma viral oncogene homolog mutation, making it resistant to many drugs. In addition to its in vitro antitumor activity, RBP was identified as a potent antagonist that substantially arrests tumor growth and invasiveness in in vivo chicken egg and mouse xenograft tumor models. Notably, histopathological analyses revealed increased immune cell infiltration and decreased Ki-67 expression, confirming the ability of RBP to inhibit tumor cell proliferation. Taken together, these findings highlight RBP as a therapeutic anticancer biomaterial capable of impeding the progression and metastasis of RAS-mutated cancers.

## Introduction

In the field of anticancer drug discovery and drug delivery system development, innovative bioactive materials with potent anticancer properties, such as polymeric antibody drug conjugates and targeted nanoparticulated anticancer drugs, are greatly needed and have garnered increasing research attention [1,2]. Advances in biomaterials science have enabled previously undruggable targets to become druggable, offering the ability to target cancers, especially cancers with mutations of rat sarcoma (RAS) protein (e.g., Kirsten rat sarcoma viral oncogene homolog [KRAS], Harvey rat sarcoma viral oncogene homolog [HRAS], and neuroblastoma rat sarcoma viral oncogene homolog [NRAS]), with enhanced precision and efficacy. The development of target-binding inhibitory peptides or proteins also represents a pivotal frontier in the evolution of cancer therapeutics, promising a new era in treatment and improvement of outcomes [3,4]. While the inhibition of the active sites of disease targets using designed small molecules such as sotorasib and adagrasib, often in combination with biomaterials, has led to some success, an increasing number of reports have indicated the emergence of resistance after active site inhibition with these treatments [5,6]. In contrast to direct inhibition, the disruption of protein–protein interactions (PPIs) of the disease target has garnered great interest in therapeutic drug discovery, especially in bioactive peptide discovery. Synthetic peptide engineering, termed peptidomics, enables the design of peptides with sufficient binding affinity for the target protein or the protein-binding factor, thereby disrupting the PPI. In this context, synthetic peptide inhibitors with submicromolar binding affinity to mutant KRAS have been used to inhibit the PPI between the intracellular RAS and its downstream signaling partner rapidly accelerated fibrosarcoma (RAF), suggesting a potential therapeutic strategy for breast cancer.

Breast cancer is the most diagnosed cancer and the second leading cause of cancer-related death among women globally, underscoring the urgent need for innovative therapeutic strategies. While conventional treatments are often used to treat estrogen receptor α (ERα)-, progesterone receptor (PR)-, or human epidermal growth factor receptor 2 (HER2/ERBB2)-expressing tumors, the development of resistance poses a significant challenge, particularly in the context of triple-negative breast cancer (TNBC), which lacks these markers [7,8]. The absence of specific drug targets in certain cancer types highlights the dependence on genotoxic chemotherapy and the unmet need for novel drugs and therapeutic targets that address resistance mechanisms [9,10]. Dysregulation of extracellular signal-regulated kinase (ERK) in TNBC, which is intricately tied to the RAS–RAF–ERK (mitogen-activated protein kinase [MAPK]) signaling pathway, underscores the necessity for innovative intervention strategies targeting these pathways [11,12]. While the downstream ERK/MAPK cascade has been a focal point for therapeutic development, challenges persist with existing strategies [13]. Studies on the RAS gene family—KRAS, HRAS, and NRAS—have revealed the pivotal role of KRAS mutations in various cancers despite their relatively low frequency in breast cancer, emphasizing the untapped potential for precision treatments targeting this mutation [14,15].

Chemical inhibition of the mutated KRAS active site often results in tumor resistance. This is due to oncogenic G13D-mutated KRAS being activated only when guanosine triphosphate (GTP) energy is absorbed. The simple chemical inhibitor does not recognize this GTP-activated KRAS (G13D), and the overall antitumor activity is limited. To overcome this limitation, the authors propose a specific PPI between mutated GTP-activated KRAS (G13D) and RAF. The binding interface between GTP-activated KRAS (G13D) and RAF can be interfered with a peptide sequence. The RAS-binding peptide (RBP) sequence was derived from the cell-penetrating domain of human β-defensin, a protein known for its host defense responses, including anti-inflammatory activity. The structure of β-defensin was analyzed, and our group was the first to report the anti-inflammatory effects of its cell-penetrating domain [16–18]. A portion of this sequence was modified to enhance its intracellular binding affinity toward KRAS (G13D), and its ability to inhibit the oncogenic RAS–RAF interaction was subsequently investigated. The aim of this study was, therefore, to demonstrate the feasibility of a peptide-based biomaterial for cancer treatment by inhibiting intracellular PPI. Peptides, proteins, and polymers can be a good candidate to modulate the therapeutic target molecular interaction, and this controlled PPI can be a new modality to treat the disease. The identification, modeling of the PPI inhibition, in vitro and in vivo anticancer activity, and biodistribution of RBP will be discussed in detail.

In this study, RBP was slightly modified to bind RAS more selectively than the parent peptide. RBP has distinct advantages over direct covalent inhibitors and may attenuate resistance development and suppress metastasis while preserving protein structures critical for efficacy. Experimental validation in MDA-MB-231 TNBC cells harboring the KRAS (G13D) mutation demonstrated that RBP effectively inhibited ERK1/2 activation and cell proliferation, migration, and colony formation. Notably, the ability of RBP to suppress tumor growth and metastasis was confirmed in chicken egg and mouse xenograft tumor models. These results suggest that RBP is a less toxic and promising therapeutic biomaterial for the treatment of KRAS-mutant cancers.

## Materials and Methods

### Peptide preparation

The RBP was synthesized via 9-fluorenylmethoxycarbonyl chemistry and solid-phase peptide synthesis, as previously described [19], in its C-terminal amide form and purified to achieve 98% purity. To evaluate the cell-penetrating ability of RBP, rhodamine B was conjugated to the N-terminus of the synthetic peptide during the final step of solid-phase peptide synthesis. Specifically, rhodamine B was introduced as the final coupling reagent prior to the cleavage of the peptide from the resin. The carboxyl group (–COOH) of rhodamine B was activated using a coupling agent (e.g., *N*-ethyl-*N*′-(3-dimethylaminopropyl) carbodiimide hydrochloride [EDC] or *N*,*N*′-dicyclohexylcarbodiimide) to react with the amine group (–NH_2_) of the peptide, forming a stable amide bond. Afterward, the peptide was cleaved from the resin using a trifluoroacetic acid-based solution, and the side-chain-protecting groups were removed. The final product was purified and characterized via high-performance liquid chromatography and mass spectrometry to confirm the success of the conjugation and the purity of the product.

### Tumor cell lines and culture conditions

The MDA-MB-231 human breast cancer cell line and HCT-116 human colon cancer cell line were obtained from the American Type Culture Collection (MA, USA). The MCF-7 human breast cancer cell line and the HT-29 human colon cancer cell line were obtained from the Korean Cell Line Bank (Seoul, Republic of Korea). The MDA-MB-231 and MCF-7 cells were cultured in Dulbecco’s modified Eagle’s medium (DMEM; Gibco, NY, USA) supplemented with 10% (v/v) fetal bovine serum (FBS; Gibco, NY, USA) and 1% antibiotic–antimycotic (A/A; Gibco, NY, USA) in a humidified 37 °C incubator supplemented with 5% CO_2_. The HCT-116 cells were cultured in McCoy’s 5A (Gibco, NY, USA) with 10% (v/v) FBS and 1% A/A in a humidified 37 °C incubator supplemented with 5% CO_2_. HT-29 cells were cultured in RPMI (Welgene, Gyeongsangbuk-do, Republic of Korea) supplemented with 10% (v/v) FBS and 1% A/A in a humidified 37 °C incubator supplemented with 5% CO_2_. All experiments were performed with mycoplasma-free cells.

### In vitro cellular internalization

A total of 1 × 10^4^ cells were plated onto glass slides (Thermo Fisher Scientific, MA, USA). Following a 24-h incubation to allow the cells to attach, the culture medium was removed, and 100 μM rhodamine-labeled peptides were added along with fresh complete medium. Following a 10-min treatment at 37 °C in a 5% CO_2_ atmosphere, the cells were rinsed with phosphate-buffered saline (PBS) and then incubated with 1 μg/ml 4′,6-diamidino-2-phenylindole for 30 min at room temperature to stain the nuclei. The cells were rinsed, and the slides were imaged on a Carl Zeiss LSM700 confocal laser scanning microscope controlled with the ZENblack software (Carl Zeiss, Oberkochen, Germany). To calculate the fluorescence intensity of the peptide-transfected population, 1 × 10^6^ cells were plated on 6-well plates in complete medium. Following peptide treatment, the cells were harvested using 1× trypsin–EDTA for 5 min, which aided in the removal of residual rhodamine-labeled peptides attached to the cell membrane surface and minimized the presence of artifacts. The fluorescence emission of rhodamine was measured using a 575/24-nm band-pass filter (FL2) on a flow cytometer (FACSCalibur, Becton Dickinson, CA, USA). The data were analyzed on the basis of 3 independent experiments in each group.

### Binding affinity of RBP for KRAS and RAF according to surface plasmon resonance assays

The RAF, GppNHp-KRAS (G13D), and integrin alpha 3 proteins in pH 4.5 sodium acetate buffer were immobilized on a dextran-covered sensor chip surface (CM5 chip). *N*-Hydroxysuccinimide (NHS) and EDC were used as cross-linkers. The remaining unreacted active groups on the dextran surface were blocked with ethanolamine. The running buffer consisted of 150 mM NaCl, 5 mM EDTA, and 0.05% Tween 20 in 10 mM Hepes, pH 7.4. A CM5 chip without immobilized protein was used as the blank surface in the Biacore assay (Biacore T100, GE Healthcare Bio-Sciences AB, Uppsala, Sweden). To determine the binding kinetics, various concentrations of GppNHp-KRAS (G13D) and RBP were injected during the association phase for 3 min at a flow rate of 10 μl/min. The dissociation phase was conducted for 5 min. The dissociation constant (KD) was determined by fitting the data obtained from different concentrations of GppNHp-KRAS (G13D) and RBP using the BIAevaluation software.

### Molecular docking and crystal structural analysis

The crystal structure of KRAS (G13D) (Protein Data Bank [PDB] ID: 8EPW) was obtained from the PDB. The structure of the RBP peptide was modeled using AlphaFold2 [20]. The modeled RBP peptide structure was docked onto the KRAS (G13D) structure using ZDOCK [21]. The peptide localized near the Mg^2+^ ion, and the guanosine 5′-[β,γ-imido]triphosphate (GppNHp or GNP) moiety of KRAS (G13D) was selected. For the selected RBP peptide/KRAS (G13D) complex, the interaction pattern was analyzed.

### Cell viability measurement via Cell Counting Kit-8 assays

The viability of the cells was determined via Cell Counting Kit-8 (CCK-8; Dojindo Laboratories, Kumamoto, Japan) assay. The cells were plated in 96-well culture plates at a density of 5 × 10^3^ cells per well and incubated for 24 h at 37 °C in a humidified incubator with 5% CO_2_. After 24 h, the cells were treated with RBP (0, 50, 100, 150, 200, 250, 300, 350, 400, 500, 700, or 1,000 μM) and incubated for 24, 48, or 72 h. After incubation, the medium was replaced with fresh medium containing 10% (v/v) CCK-8 reagent, and the cells were incubated for 2 h. Absorbance was measured at 450 nm using a microplate reader. All experiments were performed in triplicate.

### Tumor colony forming assay

MDA-MB-231 cells were plated in 24-well culture plates at a density of 1 × 10^3^ cells per well and incubated for 24 h at 37 °C in a humidified incubator with 5% CO_2_. After 24 h, the cells were treated with RBP (0, 1, 10, 50, 100, or 200 μM) and incubated for 5 d (with replacement of the medium containing the peptide every 2 d). The cells were fixed with Carnoy’s fixative (75% methanol and 25% acetic acid) for 5 min at room temperature and stained with 0.4% crystal violet for 5 min. After staining, the cells were rinsed with distilled water, and images were taken.

### Sphere formation assays

The sphere formation potential of the cells was evaluated under nonadherent culture conditions. The cells were cultured in ultralow-attachment 6-well plates (Corning Life Sciences, MA, USA) at a density of 2 × 10^3^ cells per well, since the low overall viability of primary cells prevents the use of single-cell culture methods. The cells were grown in DMEM/F12 (Invitrogen, CA, USA) supplemented with B27 serum-free supplement (1:50; Invitrogen, CA, USA), human recombinant epidermal growth factor (EGF), and 50 ng/ml human recombinant basic fibroblast growth factor (PeproTech, NJ, USA) with 100 μM peptides at 37 °C with 5% CO_2_. Fresh medium was added every 3 to 4 d, and sphere formation was monitored. The experiment was terminated on day 10, and the number of spheres (>50 μm) was quantified.

### Cell migration assay

To assess MDA-MB-231 cell migration, 2 methods were employed: a Transwell migration assay and a scratch assay. For the Transwell migration assay, 24-well Transwell plates (8-μm pore size; Costar, Corning Incorporated, Corning, NY, USA) were used to evaluate the invasive potential of the cells. In the upper chambers, MDA-MB-231 cells (5 × 10^4^ cells/well) were plated in the presence of RBP, while the lower chambers were filled with basal DMEM. Following a 6-h incubation period, nonmigratory cells were carefully removed from the upper surface of the membrane using a PBS-soaked cotton swab. The cells that successfully migrated through the membrane were fixed in methanol for 10 min, and Giemsa staining was used to visualize them. Images were captured using an Olympus DP72 camera at ×100 magnification, and the number of migrated cells in the treated samples was compared with that in the control samples. For the scratch assay, MDA-MB-231 cells (1 × 10^6^ cells) were cultured in 6-well plates until they reached confluence. The cells were rinsed with PBS and incubated overnight in serum-free medium. A sterile 200-μl plastic pipette tip was used to create a linear scratch in the center of the monolayer. The monolayers were then washed twice with PBS to remove dislodged cells and incubated with RBP for 6, 24, 48, and 72 h. Images were taken immediately after scratching and again after 6, 24, 48, and 72 h of incubation using an Olympus CKX41 inverted microscope at ×20 magnification to evaluate the distance the cells migrated from the wound edge.

### Immunoprecipitation

MDA-MB-231 cells were prepared as described for Western blot analysis, and 100 μM RBP was added and incubated for 1 h. The cells were subsequently lysed in Pierce immunoprecipitation (IP) lysis buffer (Thermo Fisher Scientific, Waltham, MA, USA) containing protease inhibitors (Sigma-Aldrich, MO, USA) for 20 min on ice. Protein lysates (500 μg) were pulled down with 2 μg of biotin primary antibody (Santa Cruz Biotechnology, Inc., Santa Cruz, CA, USA) in 500 μl of IP lysis buffer overnight and subsequently bound to A/G Plus agarose beads (Santa Cruz Biotechnology, TX, USA) for 3 h. The beads were washed 4 times with lysis buffer, pelleted, and boiled with 2× electrophoresis sample buffer. The immunoprecipitates were separated via sodium dodecyl sulfate–polyacrylamide gel electrophoresis (SDS–PAGE) and electrotransferred to a nitrocellulose membrane. Primary antibodies against RAS (Cell Signaling Technology, Beverly, MA, USA) and glyceraldehyde-3-phosphate dehydrogenase (GAPDH; Santa Cruz Biotechnology, Inc., Santa Cruz, CA, USA) were used, and bands were visualized with chemiluminescence reagents (West Pico PLUS, Thermo Fisher Scientific, MA, USA) and a ChemiDoc imaging system (Bio-Rad, Hercules, CA, USA).

### Inhibitory activity of RBP according to the RAS–GTP assay

This assay is based on the observation of RAS–GTP, not RAS–guanosine diphosphate (GDP), which binds to RAF-1 with high affinity [22] and was performed with an Active RAS Detection Kit (Cell Signaling Technology, MA, USA) according to the manufacturer’s protocol. Briefly, starved cells were treated with RBP at various concentrations for 2 h, after which they were stimulated with 50 ng/ml EGF for 10 min to activate epidermal growth factor receptor (EGFR) signaling. The cells were subsequently lysed, and protein lysates (500 μg) from the cells and glutathione *S*-transferase (GST)–RAS-binding domain (RBD)–RAF were incubated with beads to allow binding. The beads were washed 3 times with lysis buffer, pelleted, and boiled in 2× electrophoresis sample buffer. The proteins were separated via SDS–PAGE and electrotransferred to a nitrocellulose membrane. Primary antibodies against RAS (Cell Signaling Technology, Beverly, MA, USA) and GST (Abcam, Cambridge, MA, USA) were used, and bands were visualized with chemiluminescence reagents (West Pico PLUS, Thermo Fisher Scientific, MA, USA) and a ChemiDoc imaging system (Bio-Rad, Hercules, CA, USA).

### Western blot analysis

Protein expression was confirmed via Western blot analysis. Briefly, 9 × 10^5^ cells were cultured in 100-mm-diameter dishes. When they reached 90% confluence, the cells were starved for 18 h in serum-free medium. After starvation, the cells were treated with various concentrations of RBP (10, 50, 100, 150, 200, and 250 μM) for 6 h and then treated with 50 ng/ml EGF for 10 min. At the end of the culture period, the cells were lysed for 20 min in cold lysis buffer on ice. Protein concentration was measured using bicinchoninic acid protein assays (Thermo Fisher Scientific, MA, USA). Equal aliquots of protein (40 μg) were boiled for 7 min in 5× sample buffer and separated by 10% SDS–PAGE. Proteins were transferred onto nitrocellulose membranes, followed by washing with Tris-buffered saline (TBS) containing 0.1% Tween 20 (TBST). The membranes were then blocked for 60 min at room temperature with 5% skim milk in TBST. Following the washing procedure, the membranes were incubated with primary antibodies (RAS, phospho-ERK1/2, ERK1/2, phospho-RAF proto-oncogene serine/threonine-protein kinase [phospho-c-RAF; S338], c-RAF [Cell Signaling Technology, Beverly, MA, USA], and GAPDH [Santa Cruz Biotechnology, Inc., Santa Cruz, CA, USA]) diluted in TBST containing 5% skim milk at 4 °C for 18 h. Following 3 additional washes, the membranes were incubated with a secondary antibody (horseradish peroxidase-conjugated goat anti-rabbit immunoglobulin G, diluted 1:3,000 in 5% skim milk) for 60 min at room temperature. The membranes were subsequently washed 3 more times, and protein bands were detected using chemiluminescence reagents (West Pico PLUS, Thermo Fisher Scientific, USA).

### Antitumor activity of RBP in an in ovo tumor xenograft model

Fertilized white leghorn eggs, sourced from the Société Française de Production Avicole (St. Brieuc, France), were incubated for 9 d at 37.5 °C with 50% relative humidity. On embryonic day 9 (E9), a small hole was drilled into the air sac to lower the chorioallantoic membrane (CAM), and a 1-cm^2^ window was created in the eggshell above the CAM. The MDA-MB-231 cell line was cultivated in DMEM supplemented with 10% FBS (and 1% penicillin/streptomycin). Cells (at 90% confluency, passages 30 to 32) were detached from culture plates with trypsin/EDTA, rinsed with complete medium, and suspended in graft medium. An inoculum of 1 × 10^6^ cells was added onto the CAM of each egg. The eggs were randomly assigned to groups. On day 10 (E10), tumors were detectable. They were then treated every 2 d for 9 d (E11, E13, E15, and E17); specifically, 100 μl of the vehicle or 100 μl of the peptide (10 mg/kg) was added dropwise to the tumor. Angiogenesis was estimated by calculating the number of vessels on the CAM that irrigated tumors. Because tumors generated from MDA-MB-231 cells are hemorrhagic, images were obtained at E16 (2 d before collection to ensure that all vessels in the CAM were visible). On day 18 (E18), the upper portion of the CAM containing the tumor was removed, washed in PBS, and then directly transferred to paraformaldehyde (for 48 h for fixation). The tumors were then carefully removed from normal CAM tissue and weighed. The extent of treatment toxicity was evaluated according to the number of dead embryos after 9 d of treatment and observation of visible macroscopic abnormalities at the end point.

### RBP treatment inhibits metastasis invasion

In parallel, a 1-cm^2^ portion of the lower CAM was collected to evaluate the number of metastatic cells. Genomic DNA was extracted from the CAM and analyzed for human Alu sequences via quantitative polymerase chain reaction (qPCR) with specific primers. Calculation of the Cq for each sample, the mean Cq, and the number of metastases for each group were performed with the Bio-Rad CFX Maestro software (Bio-Rad, Hercules, CA, USA). According to the real-time PCR data markup language data standard (http://www.rdml.org), Cq is defined as the cycle at which the amplification curve reaches its maximum value (fractional PCR cycles). The Cq value is obtained in the exponential phase, where the qPCR curve is linear. This is the basic result of qPCR: lower Cq values indicate higher initial copy numbers of the target gene. When the PCR efficiency is 100%, a difference of 1 cycle between 2 reactions means that there are 2 times more copies (genes) in the reaction with the lower Cq value than in the reaction with the higher Cq value. One-way analysis of variance (ANOVA) with post hoc tests was performed on the data.

### Quantitative evaluation of immune cell infiltration

On day 18 (E18), 5 tumor samples per group (different from the tumor samples used for tumor weight determination) were collected to evaluate the infiltration of immune cells. Each tumor sample was cut to a small size (<0.5 cm^3^) and kept in 5 volumes of RNAlater solution at 4 °C. The extracted RNA was analyzed via reverse transcription qPCR with specific primers for chicken CD3 and CD4 sequences. The Bio-Rad CFX Maestro software was used to calculate the Cq values for each sample, determine the mean Cq, and quantify the relative expression of immune cell markers in each group.

### Antitumor activity of RBP in a mouse xenograft tumor model

Female BALB/c nude mice (aged 5 to 6 weeks) were utilized in these studies to establish breast cancer xenograft models. These mice were obtained from Orient Bio, Inc. (Seongnam, Republic of Korea) and maintained in the Division of Laboratory Animal Resources animal facility throughout the experimental period. The selection, management, and experimental protocols, as well as the preparation of the animals, were conducted in accordance with the guidelines approved by the Institutional Animal Care and Use Committee of Seoul National University. All experiments adhered to the animal protocol approved by the Institutional Animal Care and Use Committee at Seoul National University (protocol number: SNU-171026-1-5). MDA-MB-231 cells were cultured until they reached 80% to 90% confluency and then harvested and prepared as suspensions (3 × 10^6^ cells/100 μl in PBS) mixed with Matrigel. These cell suspensions were injected into the right hind flanks of nude mice (*n* = 8). Once the tumor volume reached approximately 50 mm^3^, the mice were administered RBP (10 mg/kg) via intraperitoneal injection 3 times per week for 6 weeks. The control mice received PBS injections. The mice were weighed, and their tumor volumes were measured 3 times per week using Vernier calipers, starting on the first day of treatment. The tumor volume was calculated using the formula tumor volume = *A* × *B*^2^ × 0.52, where *A* represents the longest tumor diameter and *B* represents the shortest diameter [23]. After 6 weeks, the mice were euthanized in a sealed chamber with CO_2_ gas for 5 to 10 min, and their solid tumors were harvested and fixed in 4% paraformaldehyde for subsequent analysis via immunohistochemistry (IHC).

### In vivo blood concentration of RBP

To enable fluorescence imaging, RBP was conjugated with Cyanine5.5 (Cy5.5; Lumiprobe, Hunt Valley, MD, USA) using the NHS ester labeling method. Briefly, Cy5.5–NHS ester was dissolved in an amine-free dimethylformamide solution and added to a 0.1 M sodium bicarbonate buffer (pH 8.3 to 8.5) containing RBP. The reaction mixture was incubated at room temperature for 4 h with gentle mixing. After conjugation, the Cy5.5-labeled RBP (Cy5.5–RBP) was purified to remove unreacted dye and other impurities. To assess the time-dependent blood concentration of RBP, Cy5.5–RBP was administered to mice via intraperitoneal or intravenous injection. Blood samples were then collected 0.5, 1, 2, 4, 6, and 24 h postinjection. The fluorescence of each sample was measured using IVIS imaging, allowing for the determination of Cy5.5–RBP concentrations in the bloodstream over time.

### In vivo tissue distribution of RBP

The in vivo distribution of Cy5.5–RBP was examined following the intraperitoneal injection of Cy5.5–RBP. MDA-MB-231 cells were inoculated into the right hind flank of nude mice (3 × 10^6^ cells per mouse in 100 μl of Matrigel mixed with PBS). Once the tumors reached a volume of 50 mm^3^, the mice were administered a 100-μl intraperitoneal injection of each sample at a dose of 10 mg/kg peptide. After 24, 48, and 72 h, the mice were euthanized, and tissue samples were harvested. The distribution of peptides was analyzed via optical imaging performed with an IVIS imaging system (PerkinElmer Inc., Waltham, MA, USA).

### In vivo antitumor activity: Histological analysis

#### Ex ovo tumor xenograft model

Three tumors per group were harvested and fixed in 4% formaldehyde, washed, and then stored in 70% ethanol until needed for experiments. The tumors were cut to a standard size and placed in an embedding cassette. The paraffin blocks were sectioned at a thickness of approximately 3 to 5 μm. The cells were placed on a glass slide, stained with hematoxylin and eosin (H&E) and Ki-67, and covered in an automated machine. Pictures were taken via a microscope (Olympus BX60, serial no. 7D04032, Shinjuku, Japan) at a magnification of ×4 and a microscope camera (Olympus DP73, serial no. OH05504, Shinjuku, Japan). Picture acquisition was performed only with samples showing pathological changes and with representative animals. A total of 10 nonoverlapping fields in each section of the tumor were evaluated, and the mean is presented in the results.

#### Mouse tumor xenograft model

The tissues were fixed in 4% formalin before being embedded in paraffin. Sections with a thickness of 4 μm were deparaffinized with xylene and then rehydrated with a graded series of ethanol solutions, ranging from 95% to 100%. Antigen retrieval was achieved by heating the samples in citrate buffer (pH 6) at 95 °C in a water bath for 30 min. Endogenous peroxidase activity was blocked with a peroxidase-blocking reagent (Dako Denmark A/S, Glostrup, Denmark) for 5 min in a wet chamber. The sections were incubated with Ki-67 (Abcam ab16667, 1:100) in an EnVision FLEX antibody diluent (Dako). Antibody binding was detected through incubation with EnVision FLEX/horseradish peroxidase (Dako) for 30 min. The slides were then reacted with a diaminobenzidine solution for 2 to 3 min (EnVision FLEX diaminobenzidine + chromogen). The intensity and distribution of Ki-67-specific immunostaining were assessed using a Leica DM6B digital light fluorescence microscope following hematoxylin counterstaining.

### Statistical analysis

Statistical analysis of the results was performed using GraphPad Prism (GraphPad Software). The data are expressed as mean ± standard deviation. The results were analyzed via one-way ANOVA followed by Tukey’s multiple comparison test as a post hoc analysis. A *P* value less than 0.05 was considered statistically significant.

## Results

### Characterization of RBP and its intracellular localization in MDA-MB-231 cells

**Fig. 1.**
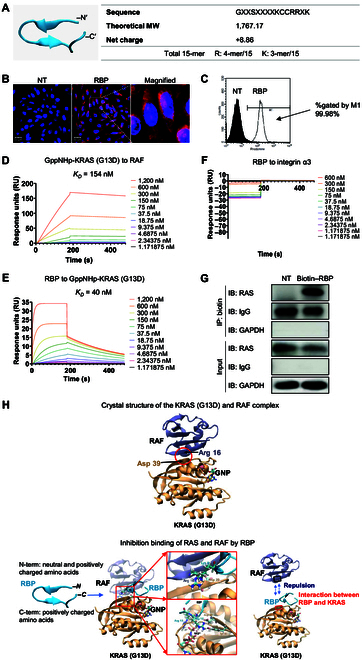
Structure and characteristics of the rat sarcoma (RAS)-binding peptide (RBP) peptide. (A) The structure of RBP and the physical properties of the RBP peptide fragments. (B) Cellular localization of rhodamine-labeled RBP in MDA-MB-231 cells. Rhodamine (red) staining is representative of RBP; the peptide was internalized throughout the cytosol and nucleus of the cell. (C) Single MDA-MB-231 cells treated with rhodamine-labeled peptides were analyzed via the CellQuest software after acquisition via flow cytometry. M1: the population containing almost all of the RBP-treated cells. (D) Sensorgrams generated by the administration of 11 concentrations of GppNHp-Kirsten rat sarcoma viral oncogene homolog (KRAS) (G13D) to surface-immobilized rapidly accelerated fibrosarcoma (RAF). (E) Sensorgrams generated by the administration of 11 different concentrations of RBP to surface-immobilized GppNHp-KRAS (G13D). RBP acts as a protein–protein interaction (PPI) inhibitor by binding to KRAS with high affinity, thereby disrupting the interaction between KRAS and RAF. (F) Sensorgrams generated by the administration of 11 different concentrations of RBP to surface-immobilized integrin alpha 3. (G) Immunoprecipitation of RBP with RAS in MDA-MB-231 cells and Western blotting of the obtained immunoprecipitates. An anti-RAS antibody was used for immunoblotting and is shown on the left of the panel. (H) The crystal structure of KRAS in complex with the RAS-binding domain (RBD) of RAF (Protein Data Bank [PDB] ID: 8EPW). The binding sites (red circle) between KRAS (G13D) and RAF are the Asp 39 residue of KRAS and the Arg 16 residue of RAF. After RBP treatment, the N-terminus of RBP interacts with the Asp 39 residue of KRAS, and the C-terminus of RBP is repelled by the Arg 16 residue of RAF. MW, molecular weight; NT, not treated; IP, immunoprecipitation; IB, immunoblotting; IgG, immunoglobulin G; GAPDH, glyceraldehyde-3-phosphate dehydrogenase; GNP, guanosine 5′-[β,γ-imido]triphosphate (GppNHp).

The structure and physical properties of RBP are shown in Fig. 1A and Supplementary Materials (Fig. S1). RBP is composed of 8 basic amino acids and has a strong positive net charge (+8.86) at pH 7.0. The positive charge, driven by arginine, lysine, and histidine residues, plays an important role in cell penetration by binding to negatively charged cell surface molecules. Among the 3 basic amino acids, the guanidine moiety of arginine facilitates integration with the hydrophobic lipid bilayer of the cell membrane. Molecular weight is crucial for assessing the effects of RBP with experimental methods such as surface plasmon resonance (SPR) and IP assays. Additionally, it ensures the accurate identification of the peptide during synthesis and purification processes (Fig. S1). The cell-penetrating activity of RBP was evaluated by treating MDA-MB-231 cells with rhodamine-labeled RBP for 10 min and observing the results via confocal microscopy (Fig. 1B). RBP was effectively internalized into the cell cytosol after 10 min of treatment. RBP directly penetrates the cell rather than undergoing endocytosis, as it was found to penetrate the cell at a low temperature. A flow cytometry assay was conducted to quantitatively assess and confirm RBP internalization (Fig. 1C). The degree of internalization of rhodamine-labeled RBP was evaluated on the basis of the mean fluorescence intensity of the RBP-treated cells. The M1 region included cells that internalized RBP, allowing comparison with the not-treated (NT) groups. Notably, 99.98% of the cells in the M1 region had internalized RBP. These confocal microscopy and flow cytometry results clearly demonstrate that RBP is effectively translocated into cells within a short treatment time.

### RBP binding affinity toward RAS and RAF

The main mechanism of action of RBP is inhibition of the interaction between intracellular RAS and RAF. The binding kinetics of RBP to intracellular RAS were measured via the BIAcore method, which revealed high-affinity binding to RAS. “KRAS” refers to the KRAS protein in general, regardless of its mutation status or bound nucleotide state. It is used in a broad context to discuss the protein family or its general biological role. When it is used by itself, it is called wild-type KRAS. “KRAS (G13D)” specifically refers to the mutant form of KRAS, in which glycine at position 13 is replaced with aspartic acid. This mutation is a common oncogenic variant and is the focus of our study. Even with mutated KRAS, the oncogenic signal does not initiate when bound to GDP. However, when KRAS binds to GTP and transitions to its active form (GTP–KRAS), the oncogenic signal is triggered. Since GTP is rapidly hydrolyzed, the nonhydrolyzable GTP analog, GppNHp, is used experimentally to mimic the active state. “GppNHp-KRAS (G13D)” is used to describe the mutant KRAS (G13D) protein bound to GppNHp, a nonhydrolyzable GTP analog. This state mimics the active, GTP-bound form of KRAS, which is critical for its interaction with RAF and downstream signaling. To assess the selectivity of this binding, we examined whether the binding affinity of RBP was specific to RAS and RAF by comparing it to the binding affinity of RBP for an unrelated receptor, integrin alpha 3. As shown in Fig. 1D, the binding affinity of GppNHp-KRAS (G13D) for RAF was measured to be 154 nM.

However, the binding affinity of GppNHp-KRAS (G13D) for RBP was 40 nM, indicating that the binding affinity of GppNHp-KRAS (G13D) for RBP is approximately 3-fold greater than that of GppNHp-KRAS (G13D) for RAF (Fig. 1E). In contrast, RBP did not bind to the unrelated receptor integrin alpha 3, confirming the selective binding affinity of RBP to RAS over other proteins (Fig. 1F). This selective and specific interaction indicates that the inhibition of RAS activation by RBP is mediated solely through the targeted binding of RBP to RAS and RAF. The binding of biotin-labeled RBP to RAS in MDA-MB-231 cells was confirmed by IP, as shown in Fig. 1G. The crystal structure of the KRAS–RAF complex was obtained from the PDB (PDB ID: 8EPW); this structure indicates that KRAS (G13D) tightly binds to RAF through an ionic interaction between the Asp 39 residue of KRAS (G13D) and the Arg 16 residue of RAF. On the basis of this structural information, the interaction between the KRAS–RAF complex and RBP was analyzed through computational modeling. According to the modeling data, the positively charged Lys 9 residue of RBP causes electrostatic repulsion with the Arg 16 residue of RAF, whereas the Arg 12 and Arg 13 residues of RBP interact with the Asp 39 residue of KRAS (G13D). These computational modeling data suggest that the binding of RBP to the Asp 39 region of KRAS (G13D), followed by electrostatic repulsion between RBP and the Arg 16 residue of RAF, effectively disrupts the KRAS–RAF interaction. Consequently, the binding of RBP to KRAS (G13D) inhibits the KRAS–RAF PPI, as shown in Fig. 1H. These results support the conclusion that RBP can interact with RAS in a real cellular environment, not just with the recombinant RAS protein.

### RBP inhibits MDA-MB-231 cell proliferation, colony formation, and sphere formation

**Fig. 2.**
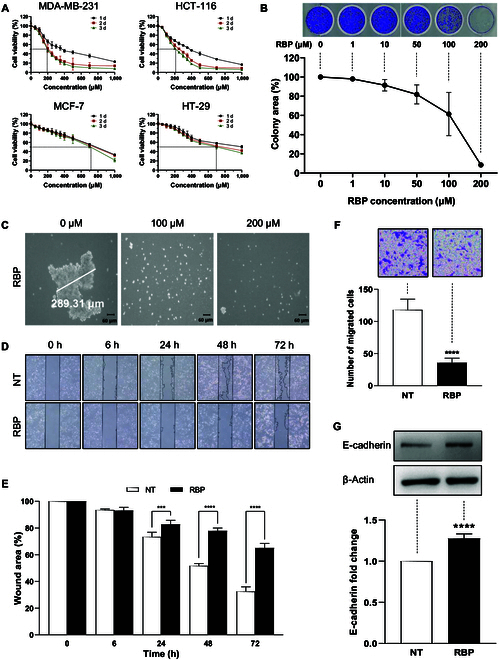
RBP inhibits the proliferation, scratch filling, and migration of MDA-MB-231 cells. (A) Cell viability of MCF-7, MDA-MB-231, HT-29, and HCT-116 cells, as evaluated via Cell Counting Kit-8 (CCK-8) assays. The percentage of viable cells was significantly decreased at considerably lower RBP concentrations in MDA-MB-231 and HCT-116 cells compared to those in MCF-7 and HT-29 cells. (B) Inhibition of in vitro clonogenicity via RBP treatment. The cells were plated at a density of 2 × 10^3^ cells/well in 24-well plates and cultured for 5 d. Finally, the cells were stained with crystal violet, photographed, and analyzed for their proliferation efficiency. (C) Spherical colonies were generated from single-cell suspensions of MDA-MB-231 cells cultured in serum-free medium supplemented with growth factors under nonadherent conditions after 3 d. However, in the RBP-treated groups, no colonies >30 μm in diameter were observed. (D) Inhibitory effect of RBP in the scratch migration assay. Scratches were made across the center of the cell monolayers via a 200-μl sterile plastic pipette tip. Images were taken using an Olympus inverted microscope both immediately after scratching and after 6 h of incubation. (E) Bar graph of the wound area. (F) Inhibitory effects of RBP on chemotactic motility were assessed via a Transwell migration assay. The cells were seeded in the upper chamber of the Transwell insert. After approximately 6 h, the MDA-MB-231 cells that invaded the membrane were counted. Transwell migration images were taken using an Olympus inverted microscope. (G) The expression of protein markers related to migration was assessed by Western blotting. The data (mean ± SD) were analyzed via analysis of variance (ANOVA). Statistical significance is indicated as ***P < 0.05 and ****P < 0.0001 (vs. NT).

The specific anticancer effects of RBP on the KRAS G13D mutation were analyzed on the basis of the rate of cell death in different KRAS cancer cell lines. To provide a control for the experiment, HCT-116 (colon cancer) cells, another KRAS G13D mutant cancer cell line, and both MCF-7 (breast cancer) and HT-29 (colon cancer) cells, which are KRAS wild-type cancer cell lines, were used. RBP treatment reduced the percentage of viable KRAS G13D mutant cells in a dose-dependent manner, as shown in Fig. 2A. However, the decrease in the percentage of viable cells among KRAS wild-type cells treated with RBP was not significant. On the basis of these results, 200 μM RBP was selected as the optimal concentration for the following experiments. Additionally, the effect of RBP on colony formation was assessed under both anchorage-dependent and anchorage-independent conditions. Under conditions favorable for cell adherence, untreated cells proliferated significantly, forming numerous large colonies after 5 d. In contrast, peptide treatment induced a dose-dependent decrease in colony area (Fig. 2B). To mimic the in vivo environment, cells were cultured on ultralow-adhesion plates to promote the formation of tumorlike spheres. These cultures were monitored over 10 d until well-defined spheres with diameters exceeding 200 μm developed. RBP was added on the first day; sphere proliferation was significantly suppressed in the RBP group compared to that in the control group, as shown in Fig. 2C. Taken together, the results of cell viability, colony formation, and sphere formation analyses demonstrated that RBP inhibited tumor growth in a dose-dependent manner.

### In vitro antimetastatic effect of RBP on MDA-MB-231 cells

To assess the antimetastatic properties of RBP in vitro, both a scratch wound migration assay and a Transwell migration assay were conducted to evaluate its inhibitory effect on chemotactic cell migration. The scratch assay revealed that RBP significantly inhibited MDA-MB-231 tumor cell migration, as shown in Fig. 2D and E. Moreover, the Transwell assay results indicated substantial suppression of the invasion of MDA-MB-231 cells treated with the peptide. Specifically, cell invasion was reduced by 70% in the group treated with 200 μM RBP compared to that in the NT group, as depicted in Fig. 2F. Western blot analysis revealed a significant increase in the expression of E-cadherin, a key marker of cell migration, in RBP-treated MDA-MB-231 cells compared to that in the NT group (1.28 ± 0.06, *P* < 0.001; Fig. 2G). These findings demonstrate that RBP inhibits cell migration and has a potent antimetastatic effect on tumor cells.

### In vitro antitumor effect: RBP inhibits the RAS–MAPK pathway

**Fig. 3.**
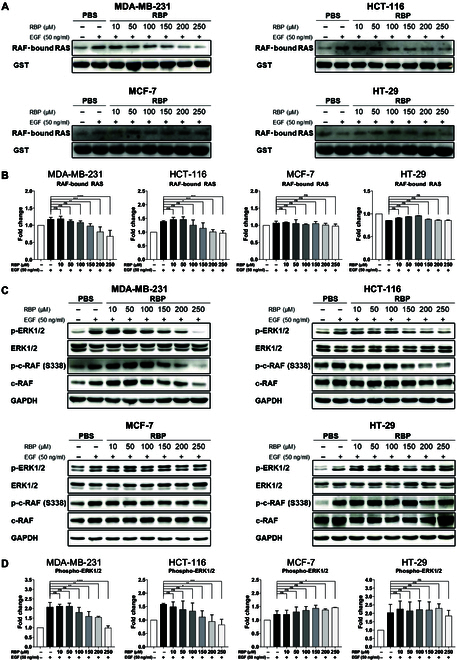
Inhibition of the expression of epidermal growth factor receptor (EGFR) signaling markers and RAF bound to RAS. (A) The expression level of RAF-bound RAS was reduced in a concentration-dependent manner by RBP in both MDA-MB-231 and HCT-116 cells. (B) Representative density analysis results for RAF-bound RAS experiments. RAF-bound RAS is expressed as a ratio of the glutathione *S*-transferase (GST) intensity values (mean ± SD; RAF-bound RAS/GST, *n* = 3). (C) Protein expression levels related to EGFR signaling were assessed via Western blotting. (D) Representative density analysis of phosphorylated extracellular signal-regulated kinase 1/2 (p-ERK1/2) data is shown. p-ERK1/2 expression is presented as the ratio of p-ERK1/2 intensity value to total ERK1/2 intensity value (mean ± SD; p-ERK/ERK, *n* = 3). The data (mean ± SD) were analyzed via ANOVA. Statistical significance is indicated as **P < 0.01, ***P < 0.05, and ****P < 0.0001 (vs. the epidermal growth factor [EGF]-treated control). ns, not significant; PBS, phosphate-buffered saline; c-RAF, RAF proto-oncogene serine/threonine-protein kinase; p-c-RAF, phosphorylated c-RAF.

Active RAS interacts with the RBD of RAF [14]. The level of RAF-bound RAS in 4 cell types after RBP treatment was determined via an IP assay. The detectable protein GST–RBD–RAF was used in these experiments; RBP significantly reduced the level of RAF-bound RAS in MDA-MB-231 and HCT-116 cells in a concentration-dependent manner, as shown in Fig. 3A and B. However, the reduction in the level of RAF-bound RAS induced by RBP treatment was not statistically significant in MCF-7 and HT-29 cells. To further validate the impact of RBP on the RAS–MAPK pathway, Western blotting was performed. The results demonstrated that RBP effectively inhibited the activation of RAF and the subsequent phosphorylation of ERK1/2 in MDA-MB-231 and HCT-116 cells, as shown in Fig. 3C and D. In contrast, RBP did not inhibit RAF activation or ERK1/2 phosphorylation in MCF-7 and HT-29 cells. The results of a GST pull-down assay confirmed that 150 μM RBP treatment significantly inhibited RAS–RAF binding in KRAS G13D mutant cancer cells and suppressed ERK1/2 phosphorylation.

### RBP inhibited tumor growth and metastatic invasion in the chicken egg xenograft model

**Fig. 4.**
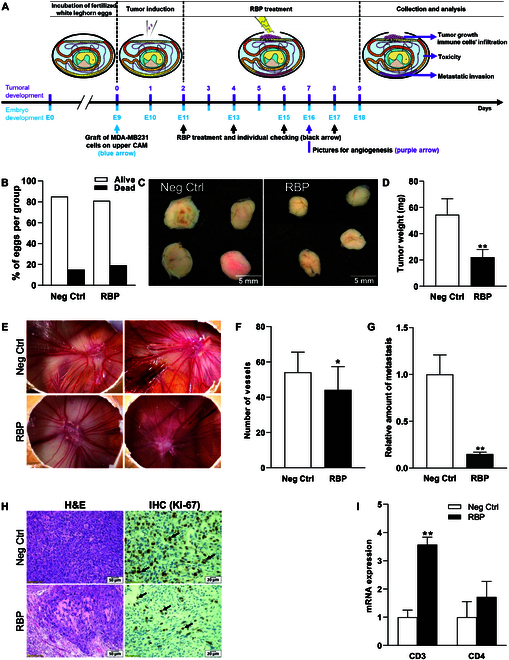
Evaluation of RBP in an in ovo xenograft model. (A) A general description of the in ovo xenograft study. (B) The percentages of dead and surviving embryos after 9 d of treatment in the different experimental groups are presented. (C) Images showing 4 tumors (ex ovo) collected from each group. (D) The mean weights (mg) of tumors on the chorioallantoic membranes (CAMs) of different experimental groups after 7 d of treatment are shown in the graphs. (E) Two representative images of tumors in the CAM (with visible vessels) from each group are presented. (F) The mean values ± SDs for the number of tumor-surrounding vessels. (G) The mean values ± SDs for metastatic cell abundance in the lower CAM of each experimental group (the arbitrary unit value for the negative control was set to 1). (H) Representative images (×10) for each group showing immune cell infiltration in tumors stained with hematoxylin and eosin (H&E) and representative images showing Ki-67-positive cells (black arrow) at ×20 magnification. (I) The expression of each immune cell markers (indicating the degree of infiltration of the cell type) were normalized to the expression of GAPDH. Mean chicken CD3 and CD4 expression levels in tumors (representing CD3-positive immune cells in tumors). The data (mean ± SD) were analyzed via *t* tests. Statistical significance is indicated as *P < 0.05 and **P < 0.01 (vs. negative control [Neg Ctrl]). IHC, immunohistochemistry; mRNA, messenger RNA.

The chicken egg xenograft model was generated via the inoculation of MDA-MB-231 cells, and the detailed experimental procedures are shown in Fig. 4A. Treatment with 10 mg/kg RBP resulted in no statistically significant difference in mortality compared to the negative control (Neg Ctrl) group, indicating that RBP does not exhibit toxicity under the tested conditions (Fig. 4B). The isolated tumors are shown in Fig. 4C; RBP treatment suppressed the growth of the MDA-MB-231 cell-derived tumors. The average tumor weight was significantly lower in the RBP treatment group than in the negative control (Neg Ctrl) group (22.10 ± 5.86 mg vs. 54.40 ± 12.22 mg in the Neg Ctrl group) (Fig. 4D, *P* < 0.01). To evaluate tumor angiogenesis, images were taken of the upper CAM containing tumors on day E16, and the number of blood vessels reaching and feeding the tumors was counted. The number of vessels was significantly reduced in the RBP-treated group (Fig. 4E and F, *P* < 0.05). In addition, the formation of microvessels was significantly suppressed in the RBP-treated group. DNA was extracted from a portion of the lower CAM on day E18, and the number of metastatic cells was assessed via qPCR analysis using specific primers for the human Alu sequence. The results revealed that RBP significantly inhibited the metastasis of MDA-MB-231 cells (Fig. 4G, *P* < 0.01). Histological analysis (H&E staining) demonstrated marked morphological differences in tumors isolated from the CAM models between the RBP-treated group and the negative control group (Fig. 4H). Further validation via IHC using an anti-Ki-67 antibody revealed reduced Ki-67 expression in RBP-treated tumor sections, demonstrating the suppressive effects of RBP on cell proliferation (Fig. 4H). Furthermore, an analysis of immune cell infiltration in tumors was performed to measure chicken biomarker expression in tumors derived from human cells. The results revealed increased infiltration of CD3- and CD4-positive immune cells in tumors following RBP treatment, as shown in Fig. 4I.

### RBP inhibited tumor growth in a mouse xenograft model

**Fig. 5.**
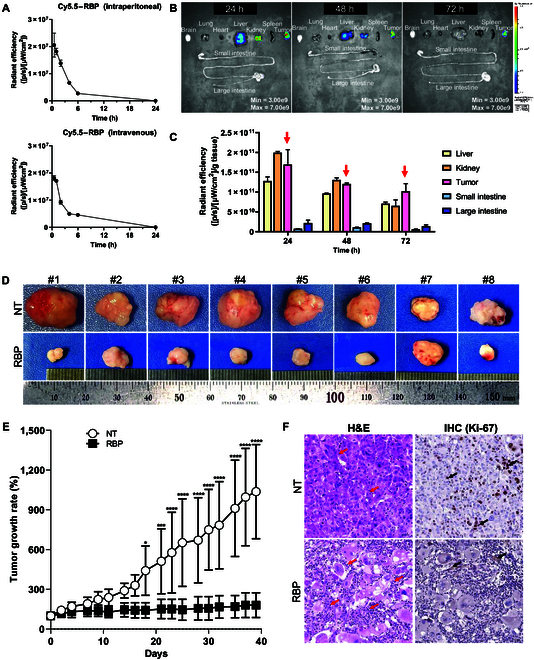
Evaluation of RBP in an in vivo xenograft model. (A) The fluorescence intensity of Cyanine5.5 (Cy5.5)–RBP in blood following intraperitoneal and intravenous administration in mice. Mice were administered 10 mg/kg of Cy5.5–RBP, and blood samples were collected 0.5, 1, 2, 4, 6, and 24 h postinjection. Concentrations of Cy5.5–RBP were quantified using IVIS. (B) The distribution of RBP in major organs. Images for the Cy5.5–RBP-treated group were acquired via IVIS 24, 48, and 72 h after intraperitoneal injection. (C) Quantification of the in vivo fluorescence intensities in tumors and other organs by normalizing to the weight of each organ. (D) Images for the comparison of tumor size in a mouse xenograft model. NT is the not-treated group, and RBP is the RBP-treated group. (E) Comparison of the tumor growth rates over 6 weeks from the start of treatment with RBP. (F) Representative images of immune cell infiltration (red arrow) in tumors stained with H&E and of proliferative cells in tumors stained with anti-Ki-67 antibody (black arrow).

RBP or Cy5.5–RBP was administered at a dose of 10 mg/kg to mice with tumors derived from MDA-MB-231 cells. To assess the time-dependent blood concentration of Cy5.5–RBP, mice were administered 10 mg/kg of Cy5.5–RBP via intraperitoneal or intravenous injection. The results showed that Cy5.5–RBP was rapidly cleared from the bloodstream through both administration routes, with minimal detectable levels remaining after 6 h (Fig. 5A). The biodistribution of Cy5.5–RBP to the tumor and major organs, including the brain, lung, heart, liver, kidney, spleen, small intestine, and large intestine, was analyzed. Strong fluorescence was observed in the tumor, liver, and kidney 24 and 48 h postinjection. However, the fluorescence intensity at 72 h decreased to 50% of the level observed at 24 h (Fig. 5B and C). Cy5.5–RBP was distributed to the tumor 24 h after injection, and the marked biodistribution of RBP into the tumor may be beneficial for the antitumor effect of RBP. The tumor images and growth rate from the initiation of RBP treatment are shown in Fig. 5D and E. A significant reduction in tumor growth was observed in the RBP-treated group compared to the not-treated group at each experimental period (*P* < 0.05).

Histological analysis revealed morphological differences between the RBP-treated group and the not-treated group (Fig. 5F). Further validation through IHC analysis using an anti-Ki-67 antibody revealed decreased Ki-67 expression in RBP-treated tumor sections, indicating the cytostatic effect of RBP (Fig. 5F).

Collectively, the results from the mouse and chicken egg tumor models were comparable, indicating that RBP is predominantly distributed within tumors and inhibits tumor growth.

## Discussion

Despite some success with chemical inhibitors in treating cancers, resistant cancers such as TNBC have been a hurdle to chemical inhibitors. As an alternative, this study demonstrated that the growth of MDA-MB-231 cells, a subtype of TNBC, was substantially inhibited by a new biomaterial, the RBP, which disrupts the RAS–RAF interaction. To achieve selective intracellular RAS–RAF inhibition, cell-penetrating biomaterials with selective binding affinity to RAS, and even GTP-activated RAS, are needed. Peptides are potential cell-penetrating biomaterials due to their functional versatility in modifying PPIs. Peptide-based anticancer biomaterials are important for increasing tumor specificity while minimizing toxicity to normal cells [24]. In particular, targeting and cell-penetrating peptides play crucial roles as targeted anticancer agents for the selective inhibition of the RAS–RAF interaction. Most RAS-targeted drugs have limited cell-penetrating capabilities, which restricts their intracellular localization. RBP, a 15-mer synthetic peptide with a cell-penetrating ability, is proposed as a new PPI inhibitor in this study. RBP has alternating arginine and lysine residues in its structure, which is different from that of arginine-rich peptides or the trans-activating transcriptional activator peptide, and these alternating residues confer high binding affinity for RAS [16]. Compared with other treatment moieties, RBP permeated cancer cells more readily (Fig. 1A to C). This cell-penetrating property of RBP suggests that it is a selective carrier for molecules with low cell-penetration properties.

Intracellular PPIs, such as the RAS–RAF interaction, play a crucial role in cell survival, as the related protein complexes facilitate signal transduction and transcription factor binding to promoters [25]. Small molecules have been developed to inhibit these PPIs and can efficiently cross the cell membrane to regulate the activity of intracellular proteins. However, tumor cells can easily acquire resistance to small-molecule PPI-targeting drugs because they cannot fully cover the large surfaces of proteins involved in PPIs. This limitation leads to the low selectivity and decreased efficacy of small-molecule PPI-targeting drugs [26]. In contrast, biologics developed to inhibit PPIs can bind to larger protein surfaces with high selectivity, but they exhibit poor cell permeability due to their large molecular size [27]. Peptides, however, share the advantages of both small molecules and biologics, as they exhibit both cell permeability and high selectivity, allowing them to cover a broad protein surface [28].

RBP is based on the cell-penetrating domain of human β-defensin 3, a protein within the innate immune defense system. This domain was modified to enhance interaction with KRAS while retaining its intrinsic membrane-penetrating properties. Studies have reported that substances with anti-inflammatory properties can also exhibit anticancer effects [29,30]. Based on these findings, in this study, we investigated the anticancer potential of RBP by applying it to specific KRAS-mutant cancer cells. This finding supports the hypothesis that RBP’s anticancer effects are mediated by its ability to target and disrupt the KRAS–RAF interaction, a critical pathway in KRAS-mutant cancers.

Our study demonstrated that RBP binds to RAS proteins and effectively disrupts the interaction between RAS and RAF (Fig. 1D and E). This disruption is critical because RAF is activated upon its association with RAS, and the inhibition of this interaction is critical for achieving an anticancer effect. The modeled RBP peptide structure was docked onto the KRAS (G13D) structure via ZDOCK. The docking results indicated that the positively charged Lys 9 of RBP causes electrostatic repulsion of RAF Arg 16, whereas Arg 12 and Arg 13 of RBP interact with KRAS Asp 39 (Fig. 1H). These interactions suggest that RBP binding disrupts the KRAS–RAF interaction through electrostatic repulsion. SPR data revealed that RBP acts as a competitive inhibitor of RAS–RAF complex formation (Fig. 1D and E). The binding affinity of GppNHp-KRAS for RAF (KD = 154 nM) was significantly lower than that for RBP (KD = 40 nM). IP experiments further confirmed that RBP binds to RAS and disrupts its interaction with RAF (Fig. 1G). In cell lysate experiments, GTP–RAS formed complexes with the recombinant RBD (the RAF-binding domain of RAS). However, RBP treatment inhibited this interaction, reducing the amount of RAF-bound RAS detected (Fig. 3A and B). Together, these results demonstrate that RBP binds to RAS and RAF with high affinity and effectively inhibits their interaction, thereby blocking downstream signaling critical for tumor progression (Fig. 3C and D). These findings highlight the therapeutic potential of RBP as a targeted inhibitor for KRAS-mutant cancers.

On the basis of the ability of RBP to penetrate the cell membrane and bind to the RAS–RAF complex, the anticancer effects of RBP in terms of cell proliferation were evaluated. Many direct covalent inhibitors targeting RAS have shown limited therapeutic effectiveness in cancer cells. The reason is that sustained administration of these covalent agents can lead to secondary mutations in RAS proteins, ultimately resulting in drug resistance. MDA-MB-231 cells are known for their high degree of drug resistance and often exhibit limited therapeutic responses [26]. Our experiments revealed that RBP effectively suppressed the proliferation of MDA-MB-231 cells in a concentration-dependent manner (Fig. 2A and B). Notably, the inhibitory effect of RBP on TNBC cells was maintained for 3 d, indicating that RBP induced low drug resistance in TNBC cells. MDA-MB-231 cells, characterized by their CD44+/CD24− stem cell-like properties and sphere-forming capabilities [31,32], exhibited reduced sphere formation upon RBP treatment (Fig. 2C). In addition, scratch and Transwell migration assays revealed significant suppression of MDA-MB-231 cell migration following RBP treatment (Fig. 2D and E). The metastatic property of cancer stem cells is closely related to epithelial-to-mesenchymal transition (EMT) [33]. EMT is characterized by the loss of E-cadherin expression and increased expression of mesenchymal markers, making tumors highly mobile and invasive. Although EMT is essential for tissue development and repair [34], EMT has also been linked to poor prognosis and the development of drug resistance, so its suppression is physiologically relevant [35]. In this study, the reduced expression level of an epithelial marker (E-cadherin) by RBP confirmed the inhibition of cell migration and tumor metastasis via the suppression of EMT (Fig. 2F and G); this mechanism may also attenuate drug resistance [36]. Collectively, these results indicate that RBP effectively inhibits cancer cell activities, including proliferation, sphere formation, and migration.

We then explored the mechanisms underlying the effects of RBP on TNBC. The function of RAS follows a cycle from an inactive GDP-bound state to an active GTP-bound state, and this cycle is tightly regulated by inactivating signals through GTPase-activating proteins and activating signals through guanine nucleotide exchange factors [37]. As RAS proteins are involved in the cell cycle, migration, proliferation, and survival of cancer cells in an active state, the regulation of RAS activity is a key mechanism for inhibiting tumor progression. MDA-MB-231 cells have a point mutation in KRAS, a member of the RAS protein subfamily. This mutation occurs at the 13th amino acid residue of KRAS, where a glycine residue is replaced with an aspartic acid residue. Mutated KRAS maintains an active state and reduces the ratio of inactive to active forms. Owing to the expression of this abnormal KRAS protein, MDA-MB-231 cells exhibit high growth and metastasis potential [14,38]. Increased active KRAS mutation in TNBC upregulates downstream signaling after RAS–RAF interaction, and specifically, the ERK protein is a key downstream protein. To examine whether RBP reduces the level of active RAS protein in MDA-MB-231 cells, we performed an active RAS IP experiment. The results confirmed that the expression of active RAS was reduced (Fig. 3A and B). This reduction in active RAS expression led to decreased RAF protein activity, followed by the suppression of ERK-related protein activity. The immunoblotting results revealed that RBP reduced phosphorylated RAF protein levels, which subsequently led to decreased phosphorylated ERK levels (Fig. 3C and D). Our study demonstrated that RBP can suppress TNBC progression by inhibiting signal transduction by KRAS through interference with the RAS–RAF interaction and by inhibiting RAF and MAPK signaling. MDA-MB-231 cells are a TNBC cell line harboring a KRAS mutation (G13D). Hyperactivation of the KRAS–RAF signaling pathway is a hallmark of this cell line, making it an ideal model for evaluating the targeting efficacy of KRAS–RAF PPI inhibitors such as RBP. The in vitro cell study aimed to demonstrate the selective antitumor activity of RBP based on this PPI inhibition. In contrast, MCF-7 cells with wild-type KRAS exhibit relatively low activation of the RAS–RAF pathway and primarily rely on the phosphatidylinositol 3-kinase–protein kinase B (PI3K–AKT) signaling pathway [39,40]. As a result, the effects of RAS–RAF PPI inhibitors are often minimal in these cells. In addition, the colon cancer cell line HCT-116 further confirmed that RBP inhibited the interaction between G13D KRAS and RAF, as compared to HT-29 cells with wild-type KRAS (Fig. 3A and B). The in vitro study demonstrated that RBP selectively targets the PPI between G13D KRAS mutation and RAF. For this reason, only MDA-MB-231 cells were used for xenograft models to demonstrate the anticancer effects of RBP.

To validate these findings in vivo, we used an in ovo chick CAM model, which mimics TNBC tumor growth and metastasis. As RBP presented selective antitumor activity in vitro in comparison with MCF-7, further in vivo studies were conducted with MDA-MB-231 cells. The live/dead ratio of RBP-treated cells was not significantly different from that of the negative control cells, indicating no toxicity after RBP treatment (Fig. 4B). In contrast, the size and weight of the xenograft tumors were reduced; these results were consistent with the observations of reduced colony and sphere numbers in the in vitro experiments (Fig. 4C and D). Treatment with RBP resulted in a reduction in the number of vessels and relative inhibition of the production of microvessels (Fig. 4E and F). In addition, RBP treatment reduced the level of MDA-MB-231 cell metastasis in the CAM model (Fig. 4G). Under physiological conditions, the invasion of immune cells is often restricted in the microenvironment of KRAS-mutant tumors, so immunotherapy is often ineffective [41]. This is further compounded by the immunosuppressive tumor microenvironment, characterized by factors such as regulatory T cells and hypoxia, which hinder immune cell infiltration and activation [42]. Additionally, immune-related adverse events and the emergence of resistance further challenge the effectiveness of immunotherapy in these contexts. Therefore, increased infiltration of immune cells, such as CD3+ and CD4+ cells, into the tumor microenvironment can improve the survival of cancer patients [43,44]. Our study revealed that RBP increased the messenger RNA expression of CD3 and CD4 markers. Increased infiltration of CD3+ and CD4+ immune cells into the tumor microenvironment was observed, suggesting an enhanced immune response and potential therapeutic benefits, including reduced tumor growth (Fig. 4I).

Tumors actively induce angiogenesis to support rapid growth, facilitating the penetration of external substances into the tumor microenvironment. According to reports, arginine-rich peptides have been reported to enhance tumor penetration while also exhibiting angiogenesis-inhibitory effects, making them a promising focus in cancer diagnosis and therapeutic strategies [45,46]. In vivo analysis of RBP in a mouse xenograft model revealed that Cy5.5-labeled RBP preferentially accumulated in tumors (Fig. 5B). In spite of the short half-life of RBP in blood, RBP was highly distributed to the tumor 24 h postinjection and remained detectable at least until 72 h. Considering the 50% decrease in fluorescence intensity of RBP at 72 h, a dosing frequency of every-2-d was used for the antitumor efficacy tests in the xenograft mouse model. Treatment with RBP resulted in a reduction in tumor size and growth (Fig. 5D and E). Histological analysis revealed morphological differences between RBP-treated tumors and not-treated tumors, with increased immune cell infiltration in the RBP-treated group. Immunohistochemical analysis revealed a reduction in the expression of Ki-67, a tumor growth factor, in the RBP-treated group (Fig. 5F).

SPR evaluates molecular interactions between proteins and proteins, or between proteins and peptides. The concentrations of RBP or GppNHp-KRAS, ranging from 1.17 nM to 1.2 μM, were used to analyze the binding affinity at the molecular level. Cellular and animal studies represent different environments compared to SPR analysis. To determine the optimum concentration of RBP in MDA-MB-231 cells, the EC_50_ was established. RBP at concentrations of 150 to 200 μM was used to treat to MDA-MB-231 cells to demonstrate the inhibition of intracellular signaling based on the EC_50_. In general, the dose in animals is higher than the in vitro concentration when considering systemic absorption, distribution, metabolism, and elimination [47,48]. The blood volume of a 30-g mouse is approximately 1.8 ml, and 0.3 mg of RBP was intraperitoneally injected as 10 mg/kg. The molar concentration of 0.3 mg of RBP in a 30-g mouse is 94 μM, which is slightly lower than EC_50_. However, a dosage of 10 mg/kg of RBP demonstrated high localization at the tumor site, and this high localization was sufficient to suppress the tumor in the in vivo xenograft model, as shown in Fig. 5.

Drug resistance is a substantial challenge in cancer therapy, particularly with direct covalent inhibitors that target RAS. Established KRAS inhibitors, such as sotorasib and adagrasib, have shown significant clinical success, particularly in targeting the KRAS (G12C) mutation. However, these chemical inhibitors bind only to the active site pocket of KRAS in its less energetically activated state. Their EC_50_ is relatively high to exert antitumor activity (960 mg/kg/d in human dose), and KRAS often presents resistance to these chemical inhibitors by acquiring secondary mutations at other sites (e.g., G13D and Q61H) [49]. In comparison, selective PPI between GTP-bound activated KRAS (G13D) and RAF can be effectively disrupted by well-designed peptide sequences. Therefore, alternative PPIs, particularly peptide-based biomaterials, are more advantageous than direct covalent inhibitors due to their high specificity and ease of engineering. The peptides used in this study are derived from natural proteins and can be used as competitive inhibitors that mimic natural interactions and bind to natural proteins [27]. Chemical inhibition using small molecules excel at targeting well-defined pockets, but many PPIs involve broad, flat interfaces that lack such pockets, making it difficult to achieve strong and specific binding [5,26,27]. In contrast, peptides can cover larger contact areas and mimic natural interfaces, allowing them to disrupt PPIs with high specificity and affinity [28]. RBP was engineered with cell-penetrating capabilities to disrupt the KRAS–RAF interaction effectively. These properties emphasize the potential of peptides as promising therapeutic options, particularly in overcoming the limitations of direct covalent inhibitors in KRAS-targeted therapies. RBP, as a PPI inhibitor, can overcome drug resistance induced by mutations in cancer cells and can target various types of mutant KRAS. Various combination therapies with different mechanisms have been developed to overcome drug resistance, and the cell-penetrating properties of RBP, along with its ability to promote CD3 and CD4 infiltration, suggest potential synergy with immunotherapies and usefulness as a delivery vehicle. Because RBP exhibits cell-penetration ability, it can be applied as a drug delivery carrier to induce synergistic effects with other impermeable drugs, such as proteins or antibodies.

**Fig. 6.**
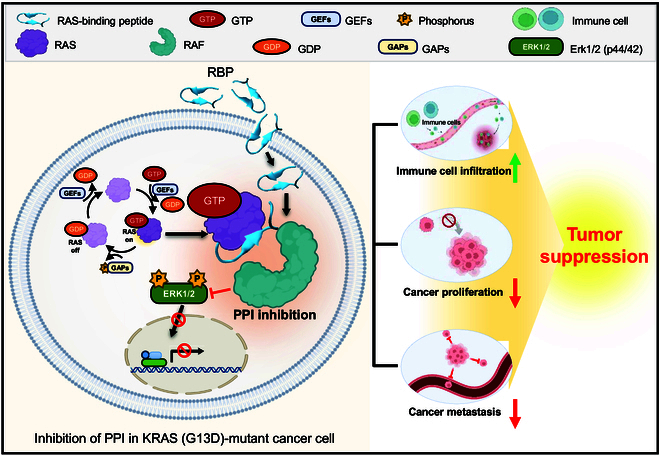
Scheme showing the mechanism by which RBP inhibits cancer cell growth. GTP, guanosine triphosphate; GDP, guanosine diphosphate; GEFs, guanine nucleotide exchange factors; GAPs, GTPase-activating proteins. This figure was created using BioRender.

Taken together, these findings indicate that RBP, after penetrating cancer cells, inhibited RAS–RAF-mediated ERK/MAPK signaling and suppressed the migration, proliferation, and growth of MDA-MB-231 cells (Fig. 6). Our findings indicate that RBP, as a PPI-inhibiting biomaterial, offers a promising alternative by preventing mutagenesis and overcoming drug resistance associated with KRAS mutations. It is noteworthy that the peptide, as a bioactive material, can be engineered to have target selectivity and thereby regulate biologic signaling.

In conclusion, we introduced RBP as a novel therapeutic biomaterial for RAS-mutated cancers that is resistant to currently available medication. The ability of RBP to penetrate cells, inhibit RAS–RAF interaction and signaling, and suppress cancer cell migration, proliferation, and metastasis highlights its potential as a versatile and effective treatment strategy for RAS-mutated cancer cells.

## Ethical Approval

All experiments in this study were conducted in accordance with ARRIVE guidelines, and all studies were performed in accordance with an animal protocol approved by the Seoul National University Institutional Animal Care and Use Committee (protocol number: SNU-171026-1-5).

## Data Availability

The data that support the findings of this study are available from the corresponding author upon reasonable request.
