# Supplementary material for: Rat Sarcoma (RAS)-Protein-Targeting Synthetic Cell-Penetrating Peptide as an Anticancer Biomaterial
Source: Biomater Res. 2025 Apr 15;29:0175. doi: 10.34133/bmr.0175 (PMC11997307; doi:10.34133/bmr.0175)
Supplement: Supplementary 1 — Fig. S1 [file bmr.0175.f1.pdf]

A

Sequence

GXXSXXXXKCCRRXK

HPLC analysis

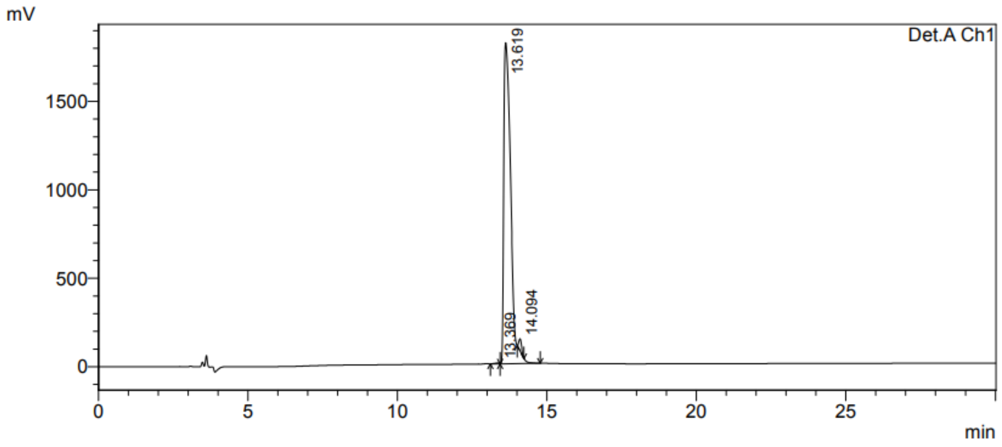

PeakTable

Detector A Ch1 214nm

Peak#	Ret. Time	Area	Height	Area %	Height %
1	13.369	65783	5788	0.223	0.307
2	13.619	28990690	1814510	98.363	96.197
3	14.094	416669	65953	1.414	3.497
Total		29473142	1886251	100.000	100.000

Mass analysis

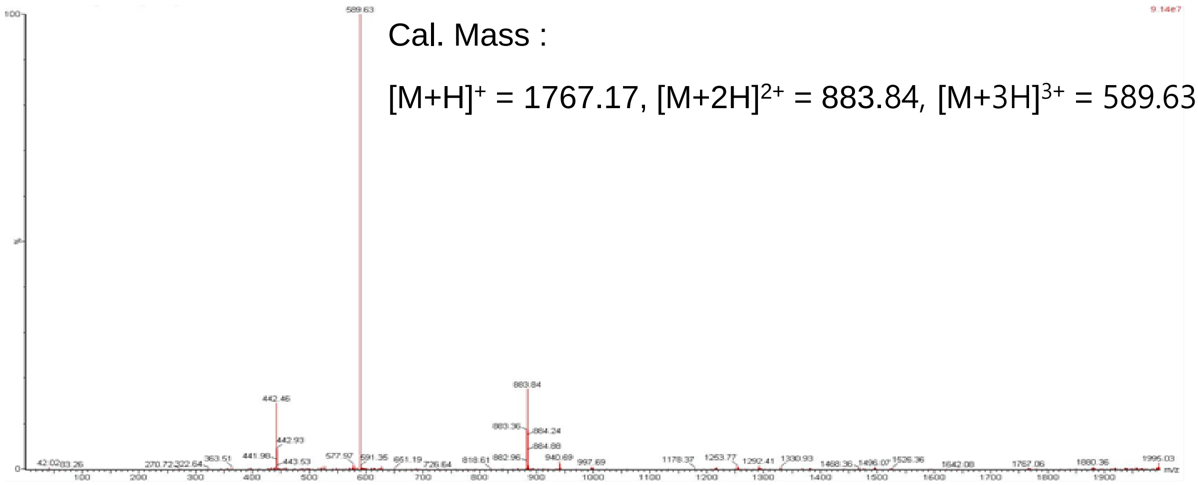

Cal. Mass :

$[M+H]^{+}$ = 1767.17, $[M+2H]^{2+}$ = 883.84, $[M+3H]^{3+}$ = 589.63

Figure S1. The sequence, HPLC analysis, and Mass analysis of RBP
